# Personalized Evidence-Based Management of Patent Ductus Arteriosus in Preterm Infants

**DOI:** 10.3390/jcdd11010007

**Published:** 2023-12-25

**Authors:** Belinda Chan, Yogen Singh

**Affiliations:** 1 Neonatology Division, Department of Pediatrics, University of Utah, Salt Lake City, UT 84113, USA; belinda.chan@hsc.utah.edu; 2 Department of Radiology and Imaging Science, University of Utah, Salt Lake City, UT 84113, USA; 3 Department of Pediatrics, Division of Neonatology, Loma Linda University School of Medicine, Loma Linda, CA 92354, USA; 4 Department of Pediatrics, Division of Neonatology, University of Southern California (USC), Los Angeles, CA 90007, USA

**Keywords:** patent ductus arteriosus (PDA), premature infant, preterm infant, medical treatment, indomethacin, ibuprofen

## Abstract

There is no universal consensus on management of patent ductus arteriosus (PDA) in preterm infants and it varies significantly worldwide, even among the clinicians within units. The decision to treat requires a thorough understanding of the clinical status of the patient, clinical evaluation of PDA, echocardiographic diagnosis, and hemodynamic impact of ductal shunt on the pulmonary and systemic circulation. In this article, updated evidence on the efficacy and adverse effects of pharmacological treatment options and expectant management are presented, while highlighting the long-term benefits of PDA treatment remains equivocal and controversial. The authors propose a schematic targeted PDA treatment approach based on gestational and chronological age for practical clinical use, and they emphasize important future directions including advancement in PDA device closure techniques, diagnostic echo-parameters, hemodynamic evaluation to assess the impact on other organs, and understanding the long-term outcomes.

## 1. Introduction

Ductus arteriosus (DA) serves a crucial role in utero by redirecting the oxygenated blood away from the fluid-filled fetal lungs, and towards the systemic circulation for optimal fetal development [1]. Typically, in term infants, DA undergoes functional constriction and closure within 1–3 days after birth, followed by tissue remodeling to ensure permanent closure [1]. However, the DA may remain persistently patent postnatally, especially in very preterm infants, and is commonly referred to as patent ductus arteriosus (PDA). Its prevalence is inversely proportional to gestational age; more than 60% of preterm infants under 28 weeks of gestational age (GA) continue to have PDA 7 days after birth or longer [2]. The left-to-right shunting through the PDA has hemodynamic consequences, especially if shunt volume is large or significant, leading to systemic hypoperfusion and pulmonary over-circulation. Prolonged exposure to hemodynamically significant PDA (hsPDA) can result in end-organ injuries such as bronchopulmonary dysplasia (BPD), necrotizing enterocolitis (NEC), intraventricular hemorrhage (IVH), acute pulmonary hemorrhage, renal failure, and even death [3,4,5,6,7,8,9]. Although there is a strong association between PDA and these co-morbidities, isolating PDA as the sole causation factor remains challenging.

PDA is one of the most extensively studied neonatal conditions. In 1978, Cotton et al. conducted a pioneering randomized controlled trial (RCT) that demonstrated that early PDA closure in preterm infants reduced the duration of mechanical ventilation [10]. Since then, over 100 RCTs and observational studies have explored the risks and benefits of PDA closure. However, despite this vast body of evidence, there remains uncertainty about who to treat, when to treat, and how to treat. Hence, there is no universal consensus on how best to treat PDA in preterm infants and there remains a lack of standardized guidelines for PDA management globally [11,12,13,14].

PDA management evolves over the years because the shifts towards more younger surviving premature infants, better diagnostic echocardiography imaging, safer treatment options and enhanced understanding of the cardiovascular physiology with hemodynamic evaluation. Firstly, the population needing PDA treatment has changed over time. As more extremely low gestational infants are surviving, PDA is more of a problem in infants of the less than 28 weeks of gestational age. The advances in echocardiography imaging have enhanced our understanding of PDA’s natural history, hemodynamic effects, and long-term outcomes. Treatment options have also progressed over time. PDA was commonly treated with medications which have equivocal efficacy for its closure and/or with PDA ligation which have associated surgical complications. Recently, there has been an increase in treating PDA with percutaneous transcatheter closure in extremely preterm infants. PDA device closure has fewer adverse effects and better outcomes as compared to surgical ligation [15]. All these factors lead to a paradigm shift in management strategy. 

This review focuses on the latest evidence and controversies surrounding PDA management in preterm infants. The authors believe that with the lack of consensus on timing and best approach to treat PDA, currently a personalized precision medicine approach is warranted.

## 2. Basis for Treatment of PDA

Before we discuss the medical management of PDA, it is important to understand the natural history of PDA, spontaneous closure of PDA, treatment options and variation in management of PDA, the evidence to treat PDA in select infants, and how to identify hemodynamically significant PDAs in these infants.

### 2.1. Natural History of PDA Spontaneous Closure

To identify the optimal timing for PDA treatment and identify the right patient for intervention, it is crucial to understand the natural history of PDA. The closure of PDA is influenced by multiple intrinsic and extrinsic factors, such as GA at birth, birth weight, chronological age (CA), prenatal conditions, PDA anatomy and morphology, blood flow across DA, signaling factors (e.g., prostaglandins), genetic predisposition, and other clinical characteristics of the infants. These factors were comprehensively summarized in the recent article by Harmick et al., shedding light on the interplay that contributes to the closure of PDA [1].

When predicting the spontaneous closure of PDA, GA and BW stand out as the most influential factors that can be assessed at the bedside and impact spontaneous closure [16]. In the most comprehensive meta-analysis comprising 99 studies and a total of 29,532 preterm infants, Klerk et al. reported that the overall closure rate was 47% by 3 days and 61% by 7 days after birth. The PDA closure rate is inversely proportional to GA (Table 1), and lower BW proportionally correlates with a reduced rate of spontaneous PDA closure in preterm infants [16].

If the DA remains patent 7 days after birth, there remains uncertainty on how long it takes for the spontaneous closure to occur in these infants. Semberova et al. followed 280 preterm infants who had not received any active PDA closure treatment and they reported that median days for PDA closure had an inverse relationship with both GA and BW as shown in Table 1 [17]. Sung et al. demonstrated that in infants of 23–28 weeks GA (*n* = 167), 95% had spontaneous closure of PDA by the time of discharge from the hospital [2].

Semberova et al. studied 56 infants who had patent DA at time of discharge from hospital and reported that 42% of cases (n = 24) had a spontaneous closure of PDA before their first follow-up appointment, 2 infants required surgical ligation or device closure, and 30 cases (56%) remained asymptomatic from PDA perspective during their follow-up. Overall, the spontaneous closure rate of PDA was 95% by 1 year of age [17]. In another study involving follow-up of 83 infants with GA of 26–30 weeks, Nielsen et al. reported a spontaneous PDA closure rate of 66% within the first year, 72% by the second year, 80% from 2–5 years. In total, 85% of infants with a diagnosis of PDA at discharge from hospital had a spontaneous closure within 5 years [18]. The risk factors associated with failure of spontaneous closure of PDA included the large size of the PDA, left atrial (LA) enlargement, and the presence of pulmonary hypertension [18].

### 2.2. Different Approaches in PDA Management

The likelihood of spontaneous closure of PDA in the vast majority of cases has sparked considerable debate on the need for PDA treatment in preterm infants. Currently, two approaches for PDA management in preterm infants are being described: (1) The conservative approach is based upon the belief that pharmacological or procedural intervention of PDA causes more harm by interrupting the natural closure process, (2) The active approach is based upon the concerns that prolonged exposure to significant PDA shunt may lead to pathological conditions causing short- and long-term consequences. This review article presents evidence supporting each of these two conflicting arguments for the management of PDA in preterm infants.

### 2.3. Evidence to Support Conservative PDA Treatment Approach

Historically, PDA was treated with pharmacological agents (traditionally by non-steroidal anti-inflammatory drugs (NSAIDs such as ibuprofen and indomethacin), or surgical ligation soon after diagnosis of PDA. The findings from the Trial of Indomethacin Prophylaxis in Preterms (TIPP) shifted towards conservative PDA treatment approach [7]. The results of the clinical trial, involving1200 preterm infants weighing less than 1000 g, showed that prophylactic pharmacological treatment of PDA did not yield any long-term neurodevelopmental benefits, despite effectively reducing the incidence of IVH and acute pulmonary hemorrhage as compared to the control group [7]. A recent meta-analysis, summarizing 138 RCTs involving 11,856 preterm infants, also found that prophylactic pharmacological treatments effectively reduce the incidence of severe IVH, but these treatments have not improved the composite outcome of death or moderate/severe neurodevelopmental disability [19]. Similarly, a recent Cochrane review showed that prophylactic PDA pharmacological treatment did not decrease the risk of IVH, CLD, NEC, and mortality [20]. The PDA-TOLERATE trial, which investigated early routine PDA treatment within 6–14 days after birth among <28 weeks GA infants with a moderate to large PDA, revealed no reduction in the incidence of PDA ligation or prevalence of PDA at discharge compared to the control group [21]. On the contrary, the treatment group had an increased risk of late-onset sepsis, delayed time to achieve establishment of full feeds, and higher mortality among ≥26 weeks GA infants [21]. Overall, a comprehensive analysis of multiple published RCTs and observational studies published up to 2020 concluded that prophylactic or early pharmacological treatment of PDA has failed to show a reduction in mortality, BPD, NEC, and other morbidities [22,23,24]. Several recently published large RCTs, such as the Baby-OSCAR trial, the BeNeDuctus trial, and the TRIOCAPI trial, have included assessment of the hemodynamic impact of PDA but still consistently showed no long-term benefits associated with prophylactic treatment [25,26,27,28,29]. The latest BeNeDuctus RCT trial published in 2023 showed that expectant management of PDA in <28 weeks GA infants (n = 136) was non-inferior to early (<72 h) ibuprofen treatment (n = 137) in terms of a composite outcome of NEC, BPD, or death at 36 weeks GA [27].

All PDA treatments have associated risks from the intervention. A non-selective prophylactic treatment exposes a large number of infants to potential adverse effects of medications unnecessarily as PDA may close spontaneously in some of these infants [20]. Pharmacological medical treatment may lead to kidney injury and gastrointestinal bleeding [20]. Surgical PDA ligation is associated with even more serious complications, including vocal cord paralysis, post-ligation syndrome, pneumothorax, infection, or bleeding [30].

The evolving evidence has prompted significant changes towards conservative PDA management. A study conducted at 19 children’s hospitals in the USA revealed an 11% decrease in treatment of PDA from 2005 to 2014. Similarly, an extensive database encompassing over 61,000 preterm infants from 280 NICUs demonstrated a decline in the incidence of PDA diagnosis from 51 to 38%, a reduction in pharmacological treatment for PDA from 32 to 18%, and a 5% decrease in the PDA ligation rate when findings were compared between the two periods of 2011–2015 and 2006–2010 [31]. These changes have been witnessed across various countries worldwide [32,33,34].

### 2.4. Evidence to Support Active PDA Treatment Approach

The clinical trials targeting PDA closure have failed to show significant long-term benefits. However, clinical observational studies consistently showed a strong association between PDA and neonatal morbidities; as more co-morbidities have been observed within the same institution when more infants have their PDA managed conservatively over time [3,4,5,6,7,8,9]. A few explanations for these contradicting data have been proposed.

Firstly, the infants in the active PDA closure trials were heterogenous, particularly in their GA range. About 60% of the enrolled infants were beyond 26 weeks of GA whose PDA were more likely to close even without active treatment. Only fewer infants <26 weeks GA were enrolled in these trials, who are more likely to be affected by a hsPDA and less responsive to pharmacological treatment [26]. They may exhibit lower tolerance to the hemodynamic stress imposed by PDA, and a higher incidence of death or BPD compared to other GA subgroups [35]. Active treatment in the lower GA group may show more long-term benefits.

Secondly, the PDA trials analysis was based on intention-to-treat or if pharmacological treatments were given rather than actual PDA closure. Currently, pharmacological treatments report a PDA closure efficacy rate of 50–70% [19,20,26,36,37,38], hence a portion of infants in the treatment group may still have open PDA. On the other hand, infants in the control group may have open or spontaneously closed PDA. A major criticism of the RCTs is the high proportion of open-label or cross-over cases in the control arm [27,39]. About 52% (38–71%) of control patients in the 32 RCTs received PDA treatment at the treating clinician’s discretion [27]. Hence, the actual difference between the treatment and control arm in exposure duration to symptomatic PDA might have been lower than expected. The PDA-TOLERATE trial also exhibited selection bias in the enrollment process. Thirteen percent of non-enrolled patients, who were younger in GA and required more respiratory support, underwent PDA treatment as early as 5 days after birth, 3 days earlier than the infants in the treatment arm [21]. These non-enrolled infants, who received PDA treatment, had a lower incidence of BPD compared to the study patients. This may support the case for an earlier closure of PDA [40].

Lastly, around 25% of the 67 PDA trials did not provide details on how PDA was diagnosed, and 10% used clinical criteria instead of echocardiography evidence of hemodynamically significant PDA. The lack of standardized PDA diagnosis criteria raises the question of whether all enrolled infants had a moderate or large PDA, or PDAs in some cases were small and insignificant. Inconsistent diagnostic approach undermines the reliability and credibility of the study results.

The heterogenicity of RCTs generated equivocal evidence to support the active PDA closure approach. However, increasing numbers of transcatheter PDA closures in very preterm infants have provided invasive hemodynamic measurements to evaluate the impact of the PDA shunt. Cardiac catheterization data showed that the longer the duration of PDA exposure, the higher was the pulmonary vascular resistance (PVR) developed over time [41]. Prolonged pulmonary over-circulation from the PDA shunt leads to pulmonary vascular remodeling and an increase in PVR. Recently, Sathanandam et al. compared the invasively measured systolic pulmonary and systemic blood pressure in preterm infants undergoing transcatheter PDA closure [42]. They reported that infants with lower systolic pulmonary pressure were younger in chronological age (27 vs. 72 days, *p* < 0.001) and thus had a shorter exposure duration to the PDA effect. The study suggests that systolic pulmonary pressure and PVR may increase over time in the presence of PDA shunt. Infants with lower PVR recovered from the device closure procedure faster and extubated sooner, even when they were younger in chronological age at the time of intervention [42,43]. In addition to catheterization data, MRI brain imaging has shed light on the negative impact of prolonged PDA duration on cerebellar growth, and the subsequent poor neurodevelopmental outcomes even at 2 years of corrected age [44]. Brain imaging and monitoring may improve our understanding of PDA shunt’s effect on brain development. Infants with a prolonged exposure to PDA shunt also had poorer weight gain which can impair somatic and brain growth [42]. These studies provide additional strong and convincing evidence to support active PDA treatment, especially in infants with hemodynamically significant ductal shunt.

### 2.5. Defining Hemodynamically Significant PDA

A comprehensive definition of hemodynamically significant PDA (hsPDA) can help in targeting treatment in preterm infants: when to intervene and who to treat based upon the hemodynamic impact of PDA shunt. The shunt across PDA is a dynamic that extends beyond a simple binary classification of open or closed ductus arteriosus. Factors such as PDA size, flow volume, cardiovascular load, and end-organ perfusion play crucial roles in determining the clinical effects of PDA. Many literature reviews have discussed using clinical features, echocardiographic features, and patient status to define hsPDA [45]. Various PDA scoring systems have been developed to combine these features for risk stratification and identifying infants at higher risk of co-morbidities [25,46,47,48,49]. There is an urgent need for developing a universally agreed criteria with validated sensitivity and specificity for defining hsPDA, and even more importantly, which can be used easily by both pediatric cardiologists and neonatologists in their routine clinical practice.

## 3. Medical Treatment

### 3.1. Pharmacological Treatment

**Figure 1:**
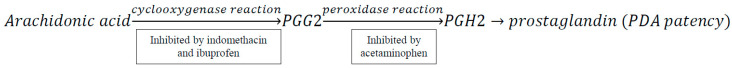
Arachidonic acid metabolism pathway.

The pharmacological treatment options for PDA closure involve the use of indomethacin, ibuprofen, and acetaminophen (also known as paracetamol). Non-steroid anti-inflammatory drugs (indomethacin and ibuprofen) inhibit prostaglandin synthesis via inhibiting cyclooxygenase (COX) reaction, while acetaminophen inhibits peroxidase action (Figure 1).

These medications for PDA treatment have been extensively studied in over 138 RCTs involving >11,000 preterm infants and multiple concluding Cochrane Reviews have 247 been published on their use in preterm infants [19,50,51]. The overall closure rate using these pharmacological agents is about 67.4% [19,36]. Indomethacin and ibuprofen have a higher efficacy around 70% (range: 57–81%) in successful closure of PDA as compared to acetaminophen (58% efficacy; range: 27–73%) [26]. Mitra et al. suggested that a high dose of oral ibuprofen was more effective in closing hsPDA vs. standard doses of intravenous ibuprofen or intravenous indomethacin, while cautiously warning the lack of improvement in other neonatal long-term outcomes after PDA treatment. The details of these medications are summarized in Table 2.

### 3.2. Expectant Treatment Approach

Expectant treatment involves non-pharmacological therapy for managing PDA in preterm infants. It is commonly employed when pharmacological treatment has failed, when infants are asymptomatic from PDA perspective, while awaiting invasive surgical ligation/device closure, or when spontaneous closure is anticipated. The common expectant treatment approach strategies include:

(1) Fluid restriction: Restricting fluid intake to 120–150 mL/kg/day was thought to decrease the size of PDA and pulmonary over-circulation [53]. However, fluid restriction carries a risk of dehydration, renal failure, and compromised nutrition intake [54]. Waal et al. recommended careful avoidance of fluid overload rather than overt fluid restriction [54]. Waal et al. summarized 11 studies on other supportive care measures [54].
(2) Diuretics: The use of diuretics, such as furosemide, may help to reduce pulmonary edema associated with PDA or BPD. However, furosemide has been linked to increased risk of ductal patency via stimulating the prostaglandin pathway [55,56]. Chlorothiazide without the prostaglandin effect can be used as an alternative but has less efficacy than furosemide [57].
(3) Optimizing ventilator settings: Optimizing the ventilator settings, such as a higher PEEP, reducing inspiratory times, and permissive hypercapnia have been suggested to temporarily stabilize respiratory status while waiting for PDA closure [54,57]. However, mechanical ventilation independently increases the risk of BPD associated with PDA, especially in infant with a PDA needing mechanical ventilation over 7–10 days [58].
(4) Targeting higher hematocrit and platelets: The maintenance of higher hematocrit and platelets levels has been suggested, but they have not shown any significant difference in successful closure of PDA and need further studies to evaluate their effects [59,60,61].

Overall, the effectiveness of these supportive measures has been disappointing and remains controversial with varying reported efficacy.

### 3.3. Timing and Treatment Approaches

The most important question remains un-answered: whether the conservative approach or the active PDA treatment strategy is the better approach to manage a hsPDA in preterm infants. Isayama et al. evaluated the observed-to-expected PDA treatment ratio and neonatal outcomes across 139 hospitals in six countries, graphically resembling a pendulum swing [34]. The extremes of both overly conservative and excessively aggressive PDA treatment approaches in preterm infants have been linked to increased risk of death or severe neurological injury. Consequently, a balanced and individualized approach between these extremes is advocated.

Many PDA management clinical guidelines have been published [1,58,62]. Besides GA, chronological age, timing to initial treatment, and desired outcomes are crucial considerations in choosing the optimal treatment approach. The Canadian Pediatric Society [62] and American Academy of Pediatrics [1] offer similar approaches under the general principles of targeted treatment:

(a) Prophylactic treatment (first 6–24 h after birth);
(b) Early symptomatic treatment with hsPDA (<7 days after birth);
(c) Late symptomatic treatment with hsPDA (≥ 7 days after birth);
(d) Expectant treatment with non-hsPDA;
(e) Discharge home with small asymptomatic PDA.

**Figure 2:**
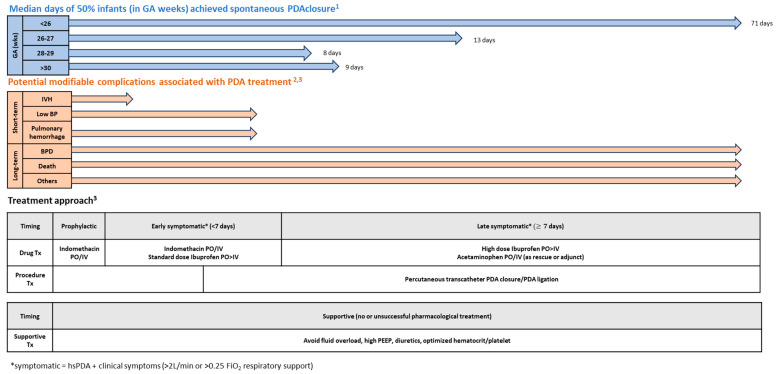
PDA medical management strategy (adapted from ^1^ Semberova 2017 [17], ^2^ Sheppahrd 2018 (https://www.eyeonprivacy.com/2018/11/fda-issues-new-draft-cybersecurity-guidance-for-medical-devices/ (Accessed date 1 November 2023)) and ^3^ Hamrick 2020 [1]).

Based on these principles, the authors propose a schematic evidence-based personalized targeted PDA treatment approach based upon gestational and chronological age as shown in Figure 2. The following section provides the explanation and evidence in support of the proposed approach and principles behind this strategy.

(a) Prophylactic treatment (within first 6–24 h after birth)

Prophylactic treatment involves using indomethacin within the first 6–24 h after birth to prevent IVH in preterm infants. Prophylactic treatment may result in overtreating 30–50% of infants who may have their PDA closed spontaneously. Hence, prophylactic treatment should be reserved for the high-risk extreme preterm infants, especially infants of <26 weeks GA, because they are unlikely to have spontaneous closure of PDA and they have a higher risk of IVH [1]. Clyman et al. have specifically recommended prophylactic treatment before 72 h after birth if the infants are <25 weeks GA, or for infants >25 weeks GA requiring mechanical ventilation within the first 72 h after birth [58].

(b) Early symptomatic treatment with hsPDA (<7 days after birth)

If PDA is not treated prophylactically, the next approach is to wait for spontaneous closure in the first 3–5 days after birth. Echocardiography is performed to confirm the presence of hsPDA by 6 days after birth. Although RCTs on PDA treatment have not supported long-term benefits, early treatment may still provide short-term benefits such as reduction in IVH, acute pulmonary hemorrhage, and hypotension [1]. Treating hsPDA under 7 days is supported by many studies that suggest an increased risk of BPD if mechanically ventilated infants are exposed to hsPDA for longer than 7–14 days [58]. Based on the published evidence, a standard dose of oral ibuprofen is recommended for treating symptomatic hsPDA during this chronological age timeframe [19]. However, more evidence is needed to support this approach in infants <26 weeks of gestation and who are receiving no or very few enteral feeds.

(c) Late symptomatic treatment with hsPDA (≥ 7 days after birth)

Beyond 7 days after birth, the consideration to treat the PDA is not only based on the presence of moderate to large PDA size, but also on the infant’s dependency on invasive mechanical ventilation. Initially, the PDA-TOLERATE trial showed early treatment showed no long-term benefits. The sub-analysis reported that infants with moderate to large PDA who were on non-invasive respiratory support had the same risk of developing BPD as those without hsPDA [58]. However, infants with persistent PDA increased the risk of BPD or death only when preterm infants needed mechanical ventilation for more than 10 days. Thus, the level of required respiratory support should be one of the determining factors for treating a hsPDA. If treatment for hsPDA is initiated beyond 7 days after birth, high-dose oral ibuprofen is preferred due to its efficacy, especially in infants tolerating enteral feeds. Adjunct or rescue treatment with acetaminophen can be used.

Chronological age at treatment initiation predicts PDA response to medications [63,64]. The likelihood of responding to pharmacological treatment of PDA decreases for every 7-day increase in chronological age at the time of treatment initiation, suggesting the need for a higher dose of medication (ibuprofen or indomethacin) to achieve the therapeutic level or earlier treatment [65]. If two courses of pharmacological therapy fail or if the medical treatment is contraindicated, percutaneous transcatheter PDA device closure or surgical ligation should be considered if hsPDA persists. In the authors’ experience, percutaneous transcatheter closure of PDA has been demonstrated to be relatively safe in preterm infants over 700 g of BW and more than 3 days after birth. It has been reported to have a high success rate, low risk of complications, and increased benefits as compared to surgical ligation. Hence it can be considered after a single course of pharmacological therapy, or at least these infants should be discussed with pediatric cardiologists as organizing transcatheter closure of PDA takes time.

(d) Expectant treatment with non-hsPDA

Expectant treatment is acceptable for low-risk infants with asymptomatic PDA, as supported by multiple RCTs [21,58]. During expectant treatment, additional evaluations may help monitor progression of PDA effects. Besides echocardiography, serum biomarkers (e.g., NT-Pro BNP), near-infrared spectroscopy (NIRS), perfusion index, and electrical cardiometry for left ventricular output measurements may be useful in monitoring PDA progression. However, the trend of these measurements is more informative than their absolute single values [65].

(e) Discharge home with small asymptomatic PDA

Finally, infants with small asymptomatic PDA can safely be discharged home with a planned cardiology follow-up. Most of the PDAs at time of discharge are likely to close spontaneously in due course but they need monitoring closely as some of them may need intervention. Semberova et al. reported that infants discharged with a hsPDA had no adverse outcomes during their follow-up [17].

## 4. Future Direction and Conclusions

To improve the management of PDA in preterm infants in future, there are several areas which can be focused on, including:

Firstly, efforts should be directed towards improving the diagnosis of symptomatic PDA and evaluating its hemodynamic impact. Echocardiography remains the gold standard diagnostic tool for the diagnosis and hemodynamic evaluation of PDA, and there is a need for standardizing the diagnostic criteria for defining hsPDA, which can be universally used by pediatric cardiologists and neonatologists in clinical practice. This will help make the practice standardized, help future research studies to study the outcomes, and, more importantly, it will help in identifying the at-risk infants for personalized precision treatment.

Secondly, understanding the impact of the duration of PDA exposure on long-term outcomes in preterm infants is crucial. More studies should be conducted to evaluate the relationship between the duration of PDA shunt exposure and various outcomes to determine the optimal timing for intervention and the potential benefits of early closure.

Thirdly, further advancement in percutaneous transcatheter closure of PDA should be explored FDA has approved use of percutaneous transcatheter closure of PDA in infants >700 g and over 3 days old. This approach has shown good efficacy and safety, even in very low birth weight infants (<700 g), as adopted by some specialized centers. In these centers, surgical ligation of PDA is reserved only for cases where transcatheter closure of PDA is not possible. Many studies have reported better outcomes and reduced complications with percutaneous transcatheter closure of PDA as compared to surgical ligation. Efforts are underway to gradually expand this procedure outside the few specialized centers. Some centers even perform percutaneous transcatheter closure of PDA at bedside in the NICU bedside using portable fluoroscopy or echocardiography to avoid the transport risk, but more studies and experience are needed to demonstrate its safety and feasibility outside the expert centers. This has been further discussed in a separate chapter on this special issue on PDA monography.

In conclusion, the management of PDA in preterm infants, especially in extremely preterm infants under 26 weeks of gestation, is a multi-faceted challenge. Both the medical treatment of PDA with pharmacological agents and invasive procedures (percutaneous transcatheter device closure and surgical ligation) have their risks and benefits. The long-term safety of the conservative treatment approach has not been established. Therefore, different approaches for PDA treatment should be adopted for different gestational ages, different chronological ages, and for those at higher risk of complications compared to low-risk infants. One size does not fit all. Hence, a personalized precision treatment approach is needed to minimize the harm and maximize the benefits, especially in high-risk extremely preterm infants.

## Figures and Tables

**Table 1:** The prevalence rate and median days of life (DOL) for the spontaneous closure of PDA.

GA (weeks)	<28		<30	<32	<37
DOL 3 [16]	34%		47%	48%	55%
DOL 7 [16]	41%		77%	63%	78%
**GA (weeks)**	**<26**	**<28**	**<30**	**>30**	
Median Day [17]	71	13	8	9	

de Klerk et al., 2020 [16] (n = 29,532); Semberova et al., 2017 [17] (n = 280).

**Table 2:** Dosing and formulation of pharmacological treatment of PDA [19,50,51,52].

	Indomethacin	Ibuprofen	Acetaminophen (Paracetamol)
Mechanism of action	Inhibits COX enzyme for prostaglandin synthesis	Same as indomethacin	Inhibits the peroxidation site of the COX enzyme for prostaglandin conversion
Standard regimen	0.1 mg/kg/dose IV × 3 doses every 24 h (for 6–12 h after birth)	10 mg/kg/dose PO or IV × 1, then 5 mg/kg/dose every 24 h × 2 doses	15 mg/kg/dose PO or IV every 6 h × 12–28 doses
High dose regimen	0.2 mg/kg/dose IV × 1 dose, then 0.1 mg/kg/dose IV every 24 h × 2 doses (for <48 h after birth) Or 0.2 mg/kg/dose IV every 24 h × 3 doses (for >2 days after birth)	15 mg/kg/dose PO or IV × 1, then 7.5 mg/kg/dose every 24 h × 2 doses Or 20 mg/kg/dose PO or IV × 1, then 10 mg/kg/dose every 24 h × 2 doses (for >5 days after birth or >28 weeks GA)	
Repeat course	May repeat up to two course	May repeat up to two course	
Formulation	IV, no commercially available PO form for neonates	Both IV and PO forms. PO is more effective than IV	Both IV and PO forms
Half-life	20 h; (12.5 h in >32-week GA; 17.5 h in <32-week GA)	16 h for PO, 30.5 h for IV. Clearance is GA and chronological age dependent	
Elimination from body	60% in urine, 40% in feces	Mostly in urine	Metabolized by liver
Adverse effects	Bleeding, negative renal effects with elevated creatinine or oliguria, bowel perforation in concurrent use of corticosteroids, further decrease mesenteric blood flow in the setting of hsPDA	Bleeding, negative renal effects (lower risk than indomethacin)	Hepatotoxicity
Efficacy and other considerations	- Prophylactic indomethacin reduces the rate of IVH (RR 0.66, 95% CI 0.53–0.82) and the need for invasive PDA closure (RR 0.51, 95% CI 0.37–0.71); but it does not show long-term benefits in reducing death or neurodevelopmental outcome (RR 1.02, 95% CI 0.90–1.15).	- Prophylactic ibuprofen has a similar effect to indomethacin in reducing the rate of IVH but lower efficacy (RR 0.67, 95% CI 0.45–1) and need for invasive PDA closure (RR 0.46, 95% CI 0.22–0.96). - In treating symptomatic PDA, both ibuprofen (RR 0.62, 95% CI 0.44–0.86) and indomethacin (RR 0.30, 95% CI 0.23 to 0.38) are more effective than placebo or no treatment. - High-dose enteral ibuprofen appears to be the most effective in PDA closure, compared to IV ibuprofen or standard dose ibuprofen [19].	- Acetaminophen has shown some efficacy in closing PDA (RR 0.35, 95% CI 0.23–0.53), though the evidence is of low certainty, especially in extremely preterm infants. - Acetaminophen can be used as an adjunct or rescue therapy if indomethacin and ibuprofen are contraindicated. - Combination of acetaminophen and ibuprofen for PDA treatment has been used to achieve a synergistic effect; however, there was no significant difference in efficacy compared to monotherapy [50].

CI confidence interval; COX cyclooxygenase; GA gestational age; h hours; IV intravenous; kg kilogram; mg milligram; PO enteral; RR relative risk.

## Data Availability

Not applicable.
